# Synchronization by Daytime Restricted Food Access Modulates the Presence and Subcellular Distribution of β-Catenin and Its Phosphorylated Forms in the Rat Liver

**DOI:** 10.3389/fendo.2017.00014

**Published:** 2017-02-06

**Authors:** Dalia Luz De Ita-Pérez, Mauricio Díaz-Muñoz

**Affiliations:** ^1^Departamento de Neurobiología Celular y Molecular, Instituto de Neurobiología, Universidad Nacional Autónoma de México, Querétaro, Querétaro, México

**Keywords:** food entrained oscillator, β-catenin variants, liver, microscopy, phosphorylation

## Abstract

β-catenin, the principal effector of the Wnt pathway, is also one of the cadherin cell adhesion molecules; therefore, it fulfills signaling and structural roles in most of the tissues and organs. It has been reported that β-catenin in the liver regulates metabolic responses such as gluconeogenesis and histological changes in response to obesity-promoting diets. The function and cellular location of β-catenin is finely modulated by coordinated sequences of phosphorylation–dephosphorylation events. In this article, we evaluated the levels and cellular localization of liver β-catenin variants, more specifically β-catenin phosphorylated in serine 33 (this phosphorylation provides recognizing sites for β-TrCP, which results in ubiquitination and posterior proteasomal degradation of β-catenin) and β-catenin phosphorylated in serine 675 (phosphorylation that enhances signaling and transcriptional activity of β-catenin through recruitment of different transcriptional coactivators). β-catenin phosphorylated in serine 33 in the nucleus shows day–night fluctuations in their expression level in the *Ad Libitum* group. In addition, we used a daytime restricted feeding (DRF) protocol to show that the above effects are sensitive to food access-dependent circadian synchronization. We found through western blot and immunohistochemical analyses that DRF protocol promoted (1) higher total β-catenins levels mainly associated with the plasma membrane, (2) reduced the presence of cytoplasmic β-catenin phosphorylated in serine 33, (3) an increase in nuclear β-catenin phosphorylated in serine 675, (4) differential co-localization of total β-catenins/β-catenin phosphorylated in serine 33 and total β-catenins/β-catenin phosphorylated in serine 675 at different temporal points along day and in fasting and refeeding conditions, and (5) differential liver zonation of β-catenin variants studied along hepatic acinus. In conclusion, the present data comprehensively characterize the effect food synchronization has on the presence, subcellular distribution, and liver zonation of β-catenin variants. These results are relevant to understand the set of metabolic and structural liver adaptations that are associated with the expression of the food entrained oscillator (FEO).

## Introduction

Daytime restricted feeding (DRF) is an accepted protocol to study the dynamic relationship between the circadian timing system and metabolic networks (1, 2). It usually involves limited access to food (a few hours each day) during a period of 2–3 weeks. A daily increase in locomotor activity before food presentation becomes an evident adaptive response after a few days of DRF conditions; this behavioral display observed is known as food-anticipatory activity (FAA) (3). DRF (2-h food access per day) involves two underlying aspects of daily physiological adjustments: (1) a circadian synchronization that shifts the phases of clock genes and (2) a hypocaloric food intake. Both aspects influence the adaptive response that allows an optimal metabolic handling of nutrients when food availability is restricted to a particular time of day (4, 5). Furthermore, a consequence of DRF is the adoption of a new coordination between the master circadian pacemaker, the hypothalamic suprachiasmatic nucleus (SCN), and peripheral oscillators such as liver, lung, adipose tissue, and heart (6, 7). Key experiments show that a variety of 24-h rhythmic responses under the DRF protocol, including the onset and maintenance of FAA, are elicited even when SCN functions are disrupted [references within Ref. (8)], which support the existence of an SCN-independent circadian timing system known as the food entrained oscillator (FEO) (9). Defining the FEO’s anatomical substrate has been elusive, in part because the existence of several FEOs in different organs and tissues (10) and the emergence of an alternative timing system that complements the SCN’s pacemaker activity (11, 12).

The liver is one of the organs that show a faster change in 24-h rhythmicity and metabolic responses under the DRF protocol (13). In this context, it has been shown that a 2-h food access during the daytime modifies (1) the circadian phase of BMAL1 and PER1 clock proteins (14, 15), (2) metabolic liver regulation (16), (3) hepatic mitochondrial activity (17), (4) ureagenesis (15), and (5) gluconeogenesis (GNG) (18). In addition to these biochemical adaptations, DRF promotes histological and ultrastructural changes in hepatocytes (19).

Conversely, β-catenin is a polyfunctional protein; it acts as a subunit of the cadherin protein complex, and hence, it regulates cell–cell adhesion properties (20). Also, it functions as a transcriptional factor acting as an effector of the Wnt signaling pathway (21), and it is a metabolic regulator that facilitates gluconeogenic activity in the liver (22). The complex role played by β-catenin is accomplished by the selective actions of phosphorylated β-catenin forms (23). Indeed, two of the best characterized phosphorylated β-catenins are as follows: (1) β-catenin phosphorylated at serine 33 (pSer33 β-catenin). This phosphorylation is carried by the serine/threonine kinase glycogen synthase kinase 3β (GSK3β) (24) after initial phosphorylation by another serine/threonine kinase, casein kinase 1 α (CK1α), at residue serine 45. Subsequent phosphorylation by GSK3β at residues T41, S37, and S33 in the *N*-terminal promotes that β-catenin be recognized by the β-TrCP E3-ligase complex, ubiquitylated, and quickly degraded by the 26 S proteasome (25). Therefore, GSK3β, CK1α, and scaffold proteins such as adenomatous polyposis coli (APC) and axin are part of the multiprotein complex called the “destruction complex” of β-catenin, whose function is to regulate cytosolic β-catenin levels. (2) β-catenin phosphorylated at serine 675 (pSer675 β-catenin). This phosphorylation is performed either by protein kinase A (PKA), a cAMP-dependent protein kinase (26), or by p21-activated kinase, a serine/threonine protein kinase (27). Phosphorylation at serine 675 enhances β-catenin transcriptional activity by facilitating the interaction between the *C*-terminal tail of β-catenin with several transcriptional coactivators, including the CREB-binding protein (28). β-catenin is linked to physiology in the metabolic zonation and metabolism of the liver due to its participation in different metabolic pathways. The importance of this functional duality consists in the preservation of liver homeostasis. It has been reported that any disruption of homeostatic balance, like the one produced by a high-fat dietary manipulation in a biological system where β-catenin is absent (a hepatocyte-specific β-catenin transgenic or β-catenin knockout mice), fosters deleterious effects on hepatocyte function and morphology (29). These effects suggest that Wnt signaling in hepatocytes is essential for the development of diet-induced fatty liver and obesity.

Evidences of relationship between β-catenin and clock proteins have come from cancer experimental models both *in vitro* and *in vivo*, where the downregulation of PER1 or PER2 proteins increased β-catenin (30) and some of its target genes such as cyclin D and C-myc. It has been proposed that previous effect is because β-catenin promotes PER1 and PER2 degradation (31). Contrary, the downregulation of β-catenin by siRNA increases the PER2 protein level in human colon cancer cells (31) and in small intestine mucosa of mice with APC mutations (APC^Min/+^). It was also observed that PER2 rhythm was lost concomitant to a reduced protein expression (31). In contrast, BMAL1, a positive regulator of the circadian clock, was demonstrated to be a transcriptional factor of the β-catenin protein and other components of the Wnt pathway (32). As a consequence, β-catenin levels in a BMAL1 null mice (BMAL1^−/−^) were decreased in comparison to wild-type mice (32). In addition, molecular mechanisms of circadian rhythmicity reside on posttranslational modifications (PTMs) of clock proteins (33), mainly phosphorylation. Most known kinases in the circadian machinery are CK1α/ε and GSK3β, which are part of the Wnt/β-catenin pathway as well. In the context of circadian mechanism, these kinases can determinate clock proteins subcellular localization, stabilization, heterodimerization, and degradation, whereas in the β-catenin context, they are involved mostly in the degradation process. Taken together, all these antecedents strongly suggest that β-catenin could be influenced by the core of the circadian molecular clock. Therefore, the purpose of this study was to explore whether DRF and the associated FEO expression could influence the 24-h rhythmicity and subcellular distribution of β-catenin variants in the rat liver.

The daily profile data in our research showed that DRF promoted (1) an enhanced presence of total β-catenins mainly in the areas adjacent to the plasma membrane, (2) a reduction in pSer33 β-catenin levels, and (3) an increase in the nuclear presence of pSer675 β-catenin. Our research also demonstrated a dynamic rearrangement in the subcellular localization of β-catenins at different times of the day. Overall, our results indicate that the β-catenin system could be part of the functional and structural adaptations that take place in the liver during the FEO expression.

## Materials and Methods

### Animals and Housing

Adult male Wistar rats weighing 200 ± 20 g at the beginning of the experiment were kept in groups in transparent acrylic cages (40 cm × 50 cm × 20 cm) and acclimated to laboratory conditions: 12:12 h light–dark cycle (lights on 08:00 hours), controlled temperature (22 ± 1°C), and free access to food (5001 rodent diet; LabDiet, St. Louis MO, USA) and water for a few days before starting the experimental procedures. Our study and the experimental protocols were approved by the Universidad Nacional Autónoma de México Institutional Animal Care and Use Committee, and all experiments were conducted in accordance to the recommendations of the Universidad Nacional Autónoma de México Institutional Animal Care and Use Committee. In addition, we took into account the International Ethical Standards reported by Portaluppi et al. (34).

### Experimental Groups

For 3 weeks, rats were randomly assigned to one of the following feeding conditions, which are similar to those reported by Davidson and Stephan (35) and Ángeles-Castellanos et al. (36):

(1) *Ad libitum* group (AL), with free access to food and water throughout the 24-h period.
(2) Daytime restricted feeding (DRF) group, which had access to food for only 2 h per day, from 12:00 to 14:00 hours. At the end of feeding conditions 1 and 2, animals were processed at 3-h intervals over a 24-h period starting at 08:00 hours. To discard the possibility that observed effects were due to the daily fasting (22 h)–refeeding (2 h) cycle in the DRF group, two additional feeding control groups were included as follows:
(3) An acute 22-h fasting group (Fa), where rats were given free access to food for 3 weeks. On the last day of the experiment, food was removed at 14:00 hours, and animals were food deprived for the next 21 h. At the end of this acute fasting (at 11:00 hours), animals were sacrificed.
(4) An acute 2-h refeeding group (Rf), where rats were left for 22 h in fasting and then refed for 2 h (from 12:00 to 14:00 hours). They were sacrificed at 14:00 hours.

Previous reports of our work group have proved the effectiveness of the DRF protocol by testing different metabolic and physiological adaptations in the rat liver such as phase shift in the daily variations of clock proteins PER1 (37, 38) and BMAL1 (14) and serum corticosterone levels (37, 38). Besides, the appearance of FAA is always associated to DRF protocol.

### Liver Sampling and Subcellular Fractionation

Animals were killed by a guillotine-like device. Livers were dissected, and a 5 g sample was processed immediately at 4°C in homogenization buffer (225 mM sucrose, 0.3 mM EGTA, 10 mM Tris-HCl, pH 7.4; 1:10 w/v), using a Potter-Elvehjem Teflon-glass homogenizer (40 rpm for 20 s). Total liver homogenate was centrifuged at 1,500 g for 15 min (Sorvall SS34 centrifuge), and the resulting pellet was isolated using the citric acid method, as reported by Reiners and Busch (39) to collect the nuclear fraction, while the resultant supernatant was decanted and centrifuged again at 10,000 g for 20 min to precipitate the mitochondrial fraction (which was discarded). The resultant supernatant was ultracentrifuged (Beckman 70Ti rotor) at 100,000 g for 70 min to obtain the microsomal fraction (which was removed) from the pellet and the cytosolic fraction from the supernatant (40). All fractions were collected, aliquoted, and stored at −70°C until further use.

### Western Blot Analyses

The total homogenate and the cytosolic and nuclear fractions were used to measure the presence of total β-catenins, pSer33 β-catenin, and pSer675 β-catenin. Total protein was quantified using the Bradford method (41). Equal amounts of protein were mixed with 2× Laemmli sample buffer (Bio-Rad Laboratories, Hercules, CA, USA) and incubated at 80°C for 10 min. The proteins were separated with 10% SDS-PAGE under reducing conditions. Subsequently, gels were transferred to nitrocellulose membranes and blocked for 1 h with 5% non-fat milk. After three washouts with 20 mM Tris-Buffered Saline and Tween 20 (TBST) (pH 7.5), membranes were incubated overnight at 4°C with the following primary antibodies (all of them diluted in TBST): rabbit anti β-catenin antibody (ab 32572) 1:5,000 dilution, rabbit anti β-catenin (phospho S33) antibody (ab 73153) (Abcam, Cambridge, MA, USA) 1:30,000 dilution, and rabbit anti β-catenin (Ser675) antibody (D2F1, Cell Signaling Technology Inc., Danvers, MA, USA) 1:1,000 dilution. The following day, all membranes were washed three times with TBST and then incubated for 2 h with the alkaline phosphatase-conjugated secondary donkey anti-rabbit antibody (sc2083, Santa Cruz Biotechnology, Dallas, TX, USA), 1:5,000 dilution. Bands were revealed using the alkaline phosphatase conjugate substrate kit (Bio-Rad Laboratories, Hercules, CA, USA). β-tubulin antibody (ab 56676) at 1:1,000 dilution was used as a loading control for homogenate and cytosolic fractions, while lamin B1 antibody (ab184115) (Abcam, Cambridge, MA, USA) at 1:10,000 dilution was used as a marker for the nuclear fraction. Quantification was done by densitometric analysis using Image J Software (42) [National Institutes of Health (NIH), USA].

### Immunofluorescence

Liver tissue was fixed in 10% formalin at 4°C for 1 week with changes every 2 days. Subsequently, the tissue was embedded in paraffin and sectioned into 7-µm slices. Liver slices were deparaffinized for 2 h at 60°C in a dry heat oven and then rehydrated in 100% xylol (10 min), 100% ethanol (5 min), 96% ethanol (5 min), 80% ethanol (5 min), and deionized water (10 min). Afterward, slices were bathed in permeabilization buffer (3.9 mM sodium citrate, 0.1% Tween 20) for 8 min and then boiled in EDTA buffer (1 mM EDTA, 0.05% Tween 20, pH 8.0) at 96°C for 1 h. Slices were blocked with 1% non-fat milk for 1 h, washed three times with TBST buffer, and incubated overnight at 4°C with the following antibodies (all diluted in TBST): rabbit anti β-catenin antibody (ab 32572) at 1:100 dilution, rabbit anti β-catenin (phospho S33) antibody (ab 73153) (Abcam, Cambridge, MA, USA) at 1:100 dilution, and rabbit anti β-catenin (Ser675) antibody (D2F1, Cell Signaling Technology Inc., Danvers, MA, USA) at 1:100 dilution.

The next day, slices were washed three times with TBST buffer and then incubated for 2 h with the secondary antibody Alexa Fluor 594 donkey anti-rabbit IgG (Invitrogen Molecular Probes Inc., Eugene, OR, USA) at 1:500 dilution. Subsequently, slices were blocked again with 1% non-fat milk for 1 h, washed three more times with TBST, and incubated overnight at 4°C with the second primary antibody, mouse anti-glutamine synthetase (GS) antibody (MAB302, Millipore Corporation, Billerica, MA, USA) at 1:300 dilution. Finally, slices were incubated for 2 h with the second secondary antibody Alexa Fluor 488 donkey anti-mouse IgG (A21202) or donkey anti-rabbit IgG (A21206) (Invitrogen, Molecular Probes Inc., Eugene, OR, USA) at 1:300 dilution. Fluorescence was visualized with both epi-fluorescence (Nikon Eclipse E600, Minato, Japan) and confocal microscopy (Zeiss Axiovert 200 LSM 510 Meta-Multiphotonic, Oberkochen, Germany), and it was quantified with Image J software (42) (NIH, USA).

### Data Analysis

The data were grouped according to experimental conditions and times (at least four rats per temporal point) and expressed as the mean ± SEM. Results were compared using the one-way and two-way ANOVA test to determine time and treatment effects, respectively. Significant differences were detected with Tukey or Sidak *post hoc* tests (*p* < 0.05). A chronobiological analysis was also carried out using the ChronosFit program (43) with the following parameters: acrophase, mesor, and percentage of rhythmicity. Finally, a Student’s *t*-test was used for the feeding condition control groups to identify significant differences between the following groups: acute fasted and refed; DRF and acute fasted (at 11:00 hours); DRF and acute refed (at 14:00 hours). All graphs and statistical analyses were performed using GraphPad Prism version 6.0 (GraphPad Software, San Diego, CA, USA).

## Results

The presence of free β-catenin in the cell cytoplasm is usually regulated across its degradation, unless β-catenin avoids the destruction complex. In this case, β-catenin increases in cytoplasm and can translocate into the nucleus. This function can be regulated by phosphorylation of a variety of kinases in the *N*-terminus or *C*-terminus of protein. First, in our experimental protocol, we evaluated β-catenin that is targeted to degradation (pSer33 β-catenin) and a β-catenin with an enhanced transcriptional activity (pSer675 β-catenin).

### DRF Protocol Decreased the Level of pSer33 β-Catenin

**Figure 1:**
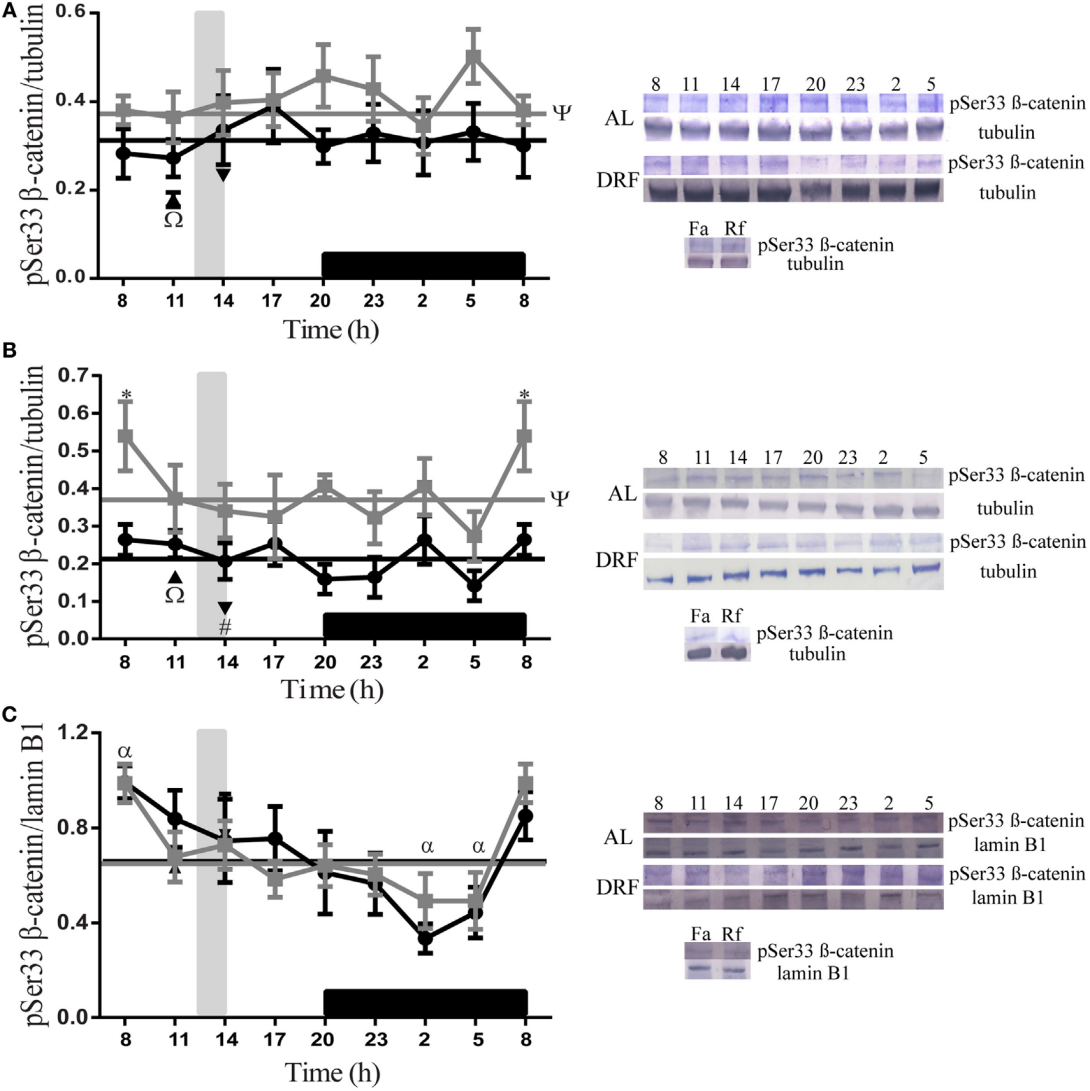
**Daily profile of pSer33 β-catenin in the rat liver under the daytime restricted feeding (DRF) protocol**. Semiquantitative western blot analysis of pSer33 β-catenin at total homogenate **(A)**, cytosolic fraction **(B)**, and nuclear fraction **(C)**. Each value was normalized using the housekeeping proteins tubulin [for **(A,B)**] and lamin B1 [for **(C)**]. A representative western blot for each condition is shown. Gray squares, AL group; black circles, DRF group; triangles, Fa group; inverse triangles, Rf group. Data are represented as the mean ± SEM (*n* = 4–5 different animals per temporal point). Horizontal lines represent the 24-h cycle average. The vertical gray bar indicates food access (12:00–14:00 hours), and the horizontal black rectangle in the *x*-axis corresponds to the dark phase. ^ψ^Significant difference between AL and DRF groups in the 24-h average (Student’s *t*-test, *p* < 0.05). *Significant difference between AL and DRF groups at the same temporal point (two-way ANOVA followed by Sidak *post hoc* test, *p* < 0.0001). ^α^Significant difference between points of the same group (one-way ANOVA followed by Tukey *post hoc* test, *p* < 0.05). ^Ω^Significant difference between Fa and Rf groups (Student’s *t*-test, *p* < 0.05). ^#^Significant difference between DRF (11:00 or 14:00 hours) versus Fa and Rf (Student’s *t*-test, *p* < 0.05).

The daily patterns of pSer33 β-catenin at different subcellular compartments of the liver are represented in Figure 1. The AL and DRF groups showed a constant presence of protein in the total homogenate and in the cytosolic fraction (Figures 1A,B) during the 24-h period. In the cytosolic fraction, the AL group at 08:00 hours presented a significant difference, two-way ANOVA hours of day: *F*(1,48) = 23.85, *p* < 0.0001, in comparison to DRF at same temporal point. Nevertheless, the DRF group showed a 16% decrease in the total homogenate and a 42% decrease in the cytosolic fraction. In the nuclear fraction, both groups displayed a gradual decrease of pSer33 β-catenin from the beginning of the light phase (08:00 hours) to the middle of dark phase (02:00 hours) (Figure 1C). In both groups, the one-way ANOVA showed significant difference. In the AL group: *F*(7,32) = 2.74, *p* = 0.02, and in the DRF group: *F*(7,32) = 2.74, *p* = 0.02. However, they did not present any rhythmicity when they were evaluated by the chronobiological analysis. The Fa group exhibited a similar expression of pSer33 β-catenin to DRF at 11:00 hours in all fractions tested, whereas the Rf group revealed a similar pattern in the total homogenate and in the nuclear fraction, but not in the cytosolic fraction; it showed a 61% reduction in pSer33 β-catenin compared to the DRF group (14:00 hours). The Fa group decreased its pSer33 β-catenin expression in the total homogenate by 23% with respect to the Rf group. In contrast, the Rf group decreased its pSer33 β-catenin expression in the cytosolic fraction by 51% with respect to the Fa group (Figures 1A–C).

### DRF Increased the Nuclear Presence of pSer675 β-Catenin

**Figure 2:**
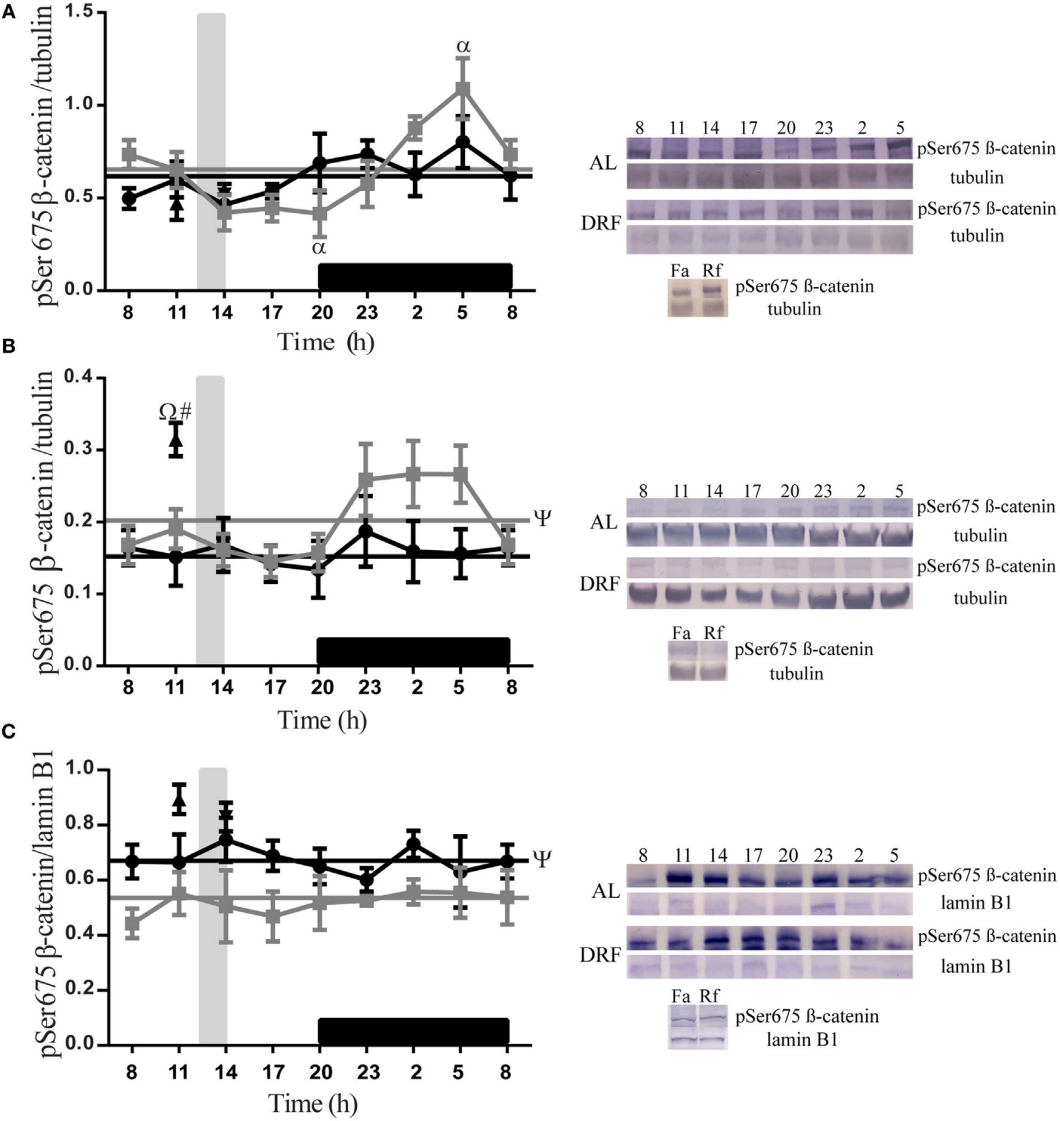
**Daily profile of pSer675 β-catenin in the rat liver under the daytime restricted feeding (DRF) protocol**. Semiquantitative western blot analysis of pSer675 β-catenin at total homogenate **(A)**, cytosolic fraction **(B)**, and nuclear fraction **(C)**. Each value was normalized using the housekeeping proteins tubulin [for **(A,B)**] and lamin B1 [for **(C)**]. A representative western blot for each condition is shown. Gray squares, AL group; black circles, DRF group; triangles, Fa group; inverse triangles, Rf group. Data are represented as the mean ± SEM (*n* = 4–5 animals per temporal point). Horizontal lines represent the 24-h cycle average. The vertical gray bar indicates food access (12:00–14:00 hours), and the horizontal black rectangle in the *x*-axis corresponds to the dark phase. ^ψ^Significant difference between AL and DRF groups in the 24-h average (Student’s *t*-test, *p* < 0.05). ^α^Significant difference between points of the same group (one-way ANOVA followed by Tukey *post hoc* test, *p* < 0.05). ^Ω^Significant difference between Fa and Rf groups (Student’s *t*-test, *p* < 0.05). ^#^Significant difference between DRF (11:00 or 14:00 hours) versus Fa and Rf (Student’s *t*-test, *p* < 0.05).

The daily patterns of pSer675 β-catenin were quantified at different subcellular compartments of the liver in both AL and DRF groups (Figure 2). In total homogenate, AL rats showed a gradual decrease in pSer675 β-catenin expression throughout the light phase (from 08:00 to 20:00 hours) and a gradual increase in the dark phase (from 20:00 to 08:00 hours) until it reached a peak at 05:00 hours, one-way ANOVA hours of day *F*(7,32) = 5.08, *p* = 0.0006 (Figure 2A). This increment favors a 24-h rhythmic pattern that presented acrophase at 4.5 h (Table 1).

**Table 1:** **Chronobiological analysis of pSer675 β-catenin in total homogenate of *Ad libitum* group**.

	MESOR	Amplitude	Acrophase (hours:minutes)	Rhythmicity (%)
pSer675 β-catenin	0.65 ± 0.24	0.30	04.55	87.43

*CHRONOS-FIT analysis was performed to evaluate the daily rhythmicity. Amplitude represents the difference between the peak (or trough) and the mean value of a wave (Mesor). Acrophase represents the time at which the peak of a rhythm occurs. The existence of a rhythmic pattern was defined by one-way ANOVA*.

In the cytosolic fraction, the presence of pSer675 β-catenin was higher in the AL group during the dark phase (Figure 2B). In the nuclear fraction, a constant expression of pSer675 β-catenin was observed throughout the 24-h period. Regarding the DRF group, a uniform presence of pSer675 β-catenin was identified in all fractions tested in the 24-h cycle. It is relevant to mention that, compared to the DRF group, the AL group showed a 21% increase in the expression of pSer675 β-catenin in the cytosolic fraction throughout the 24-h period. This daily pattern is proportionately reversed in favor of the DRF group in the nuclear fraction (Figure 2C). The Fa and Rf groups showed a similar presence of protein in the total homogenate and in the nuclear fraction when compared to the DRF group (at 11:00 and 14:00 hours). The increase of pSer675 β-catenin in the cytosolic fraction of Fa group was greater than the observed in the DRF group at 11:00 hours (52%) and in the Rf group at 14:00 hours (50%).

To discriminate among the multiple forms of β-catenin in our protocol (phosphorylated and non-phosphorylated), we also evaluated the total liver β-catenins.

### DRF Protocol Increased the Presence of Total β-Catenins

**Figure 3:**
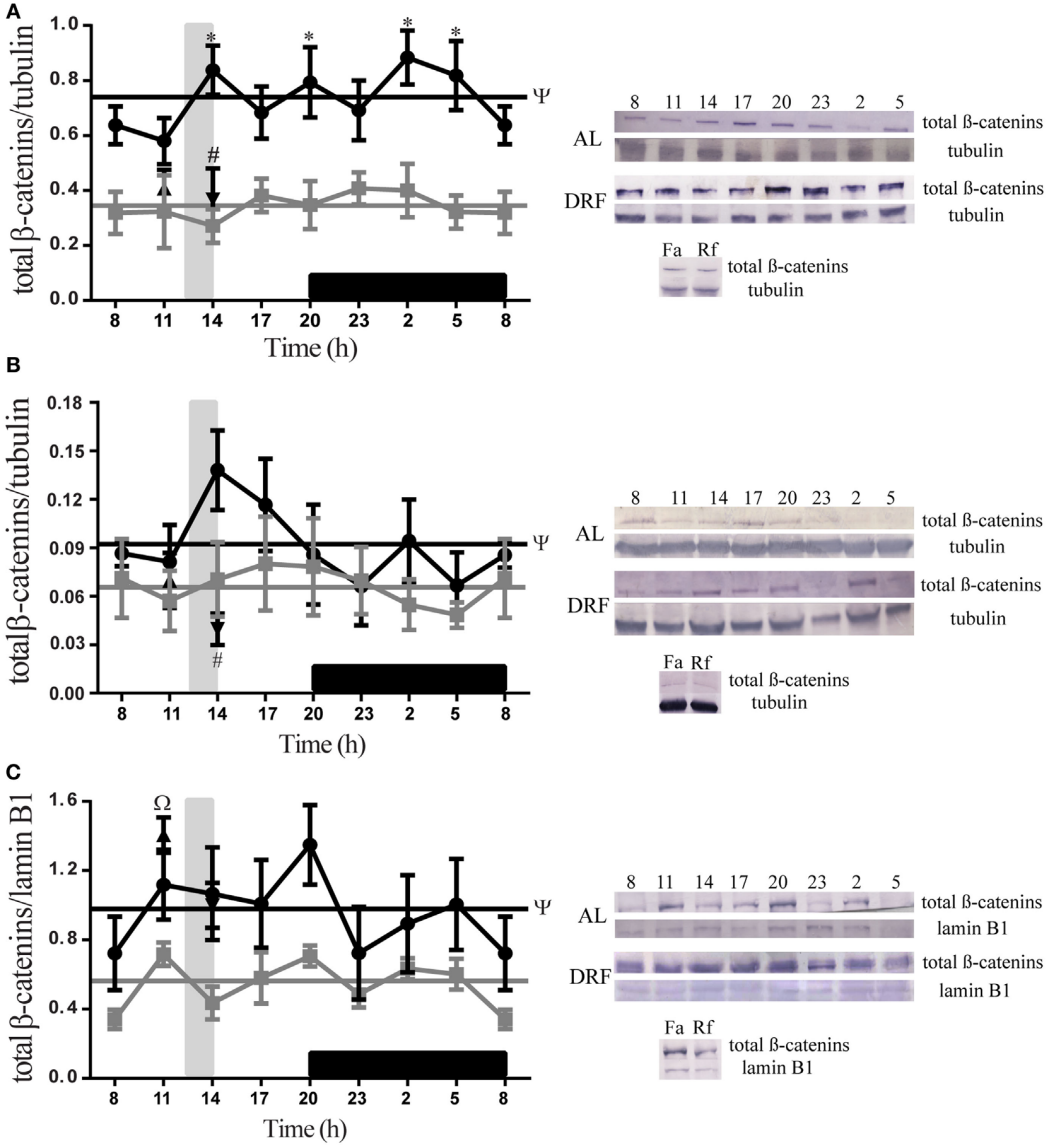
**Daily profile of total β-catenins presence in the rat liver under the daytime restricted feeding (DRF) protocol**. Semiquantitative western blot analysis of total β-catenins at total homogenate **(A)**, cytosolic fraction **(B)**, and nuclear fraction **(C)**. Each value was normalized using the housekeeping proteins tubulin [for **(A,B)**] and lamin B1 [for **(C)**]. A representative western blot for each condition is shown. Gray squares, AL group; black circles, DRF group; triangles, Fa group; inverse triangles, Rf group. Data are represented as the mean ± SEM (*n* = 5–7 animals per temporal point). Horizontal lines represent the 24-h cycle average. The vertical gray bar indicates food access (12:00–14:00 hours), and the horizontal black rectangle in the *x*-axis corresponds to the dark phase. ^ψ^Significant difference between AL and DRF groups in the 24-h average (Student’s *t*-test, *p* < 0.05). *Significant difference between AL and DRF groups at the same temporal point (two-way ANOVA followed by Sidak *post hoc* test, *p* < 0.0001). ^Ω^Significant difference between Fa and Rf groups (Student’s *t*-test, *p* < 0.05). ^#^Significant difference between DRF (11:00 or 14:00 hours) versus Fa and Rf (Student’s *t*-test, *p* < 0.05).

Figure 3 shows daily patterns of total β-catenins at different subcellular compartments of rat hepatocytes under AL and DRF conditions. The AL group showed a constant 24-h expression in all fractions tested. DRF rats did not show a 24-h rhythmic pattern. However, DRF protocol promoted significant increases in the 24-h cycle average as follows: 113% of total β-catenins in comparison to the liver homogenate in the AL group (Figure 3A), 39% in the cytosolic fraction (Figure 3B), and 75% in the nuclear fraction (Figure 3C). All three increases were greater in the light phase, when DRF animals had access to food. DRF only displayed temporal differences with the AL group, in the total homogenate at 14:00, 20:00, 02:00, and 05:00 hours; two-way ANOVA hours of day: *F*(1,88) = 69.56, *p* < 0.0001. The Fa and Rf groups showed similar total β-catenin levels to the AL group at 11:00 and 14:00 hours in the total homogenate and in the cytosolic fraction (Figures 3A,B). Nevertheless, total β-catenins levels in the nuclear fraction were similar to those in the DRF group (Figure 3C).

Due to the structural function of β-catenin at adherens junctions (AJ), we evaluated the presence of the β-catenin variants in the plasma membrane through immunohistochemistry at 11:00 hours (before food access for DRF rats), 14:00 hours (after food access for DRF rats), and 02:00 hours (in the middle of the dark phase) and under Fa and Rf conditions.

Immunohistochemical observations were used to determine how the time of day and feeding conditions influence the subcellular distribution of phosphorylated and total forms of β-catenin within hepatocytes, as well as the correlations between them.

### DRF Protocol Augmented Total β-Catenins Placed in the Plasma Membrane

**Figure 4:**
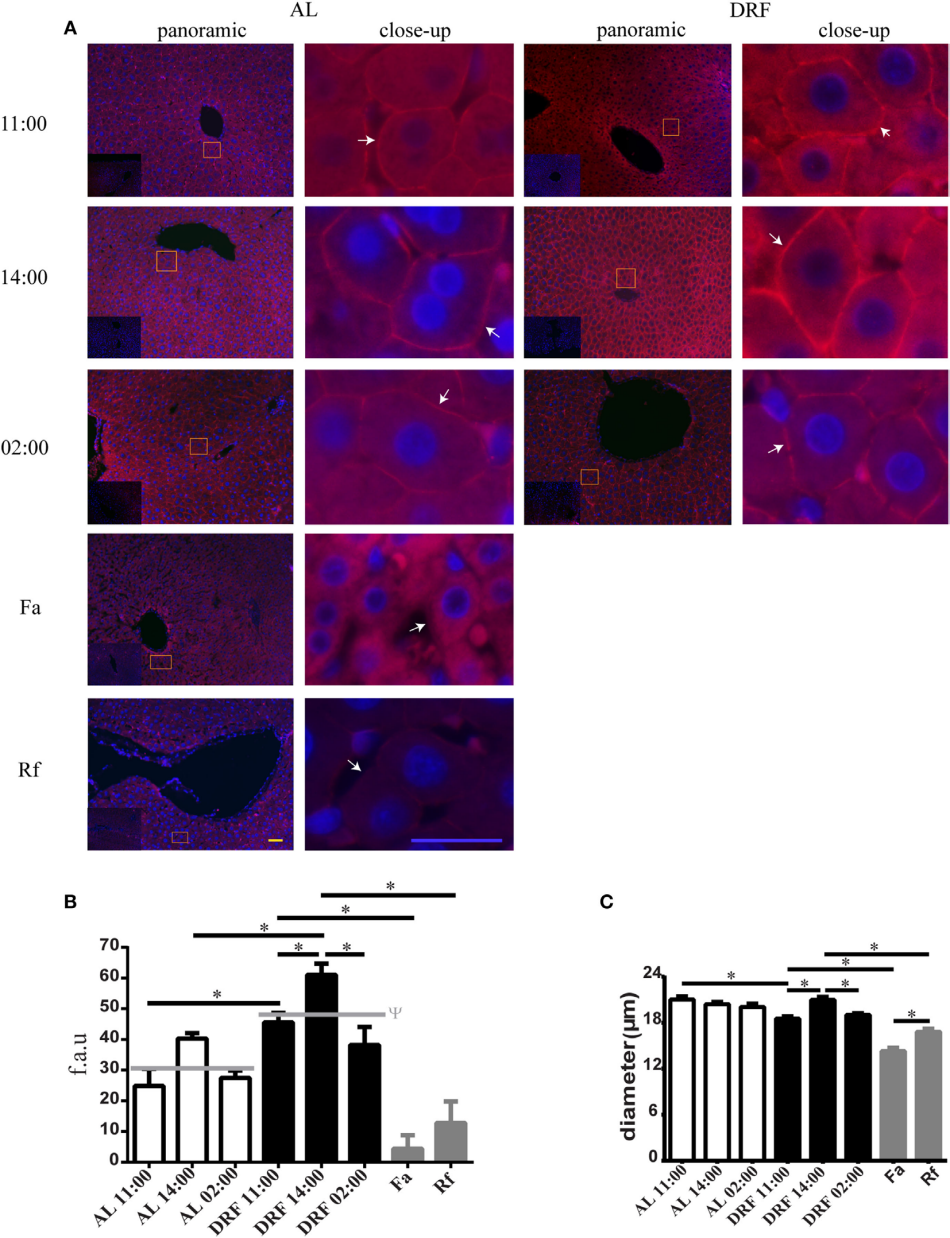
**Expression of the total β-catenins in the hepatocyte plasma membrane under the daytime restricted feeding (DRF) protocol**. **(A)** Immunofluorescence signal for total β-catenins in three different temporal points: before food-anticipatory activity (FAA) (11:00 hours), after FAA (14:00 hours), and in the middle of the dark phase (02:00 hours), as well as under Fa and Rf conditions. Both a panoramic (yellow calibration bar = 50 µm) and a close-up view (blue calibration bar = 25 µm) indicated by an orange square are shown. A negative control (primary antibody omitted) is displayed in the insert of the panoramic panels. Histograms show quantification of **(B)** total β-catenins presence in hepatocyte plasma membrane (*n* = 3 animals) and **(C)** hepatocyte diameter at same temporal points described above under AL (white bars), DRF (black bars), and Fa and Rf (gray bars) conditions (*n* = 200). Data are represented as the mean ± SEM. The horizontal gray line represents the schedules average of each condition. ^ψ^Significant difference between AL and DRF groups average (Student’s *t*-test, *p* < 0.05). *Significant difference between AL and DRF groups at the same temporal point (Student’s *t*-test, *p* < 0.05). f.a.u, fluorescence arbitrary units.

The AL group showed the presence of similar total β-catenins in all temporal points mentioned above, whereas the DRF group showed an enhanced expression of total β-catenins at 11:00 and 14:00 hours (Figures 4A,B). In contrast, the Fa and Rf groups showed the lowest expression of total β-catenins in the plasma membrane. To discover whether the expression of total β-catenins in the plasma membrane could be correlated with hepatocyte size, we measured hepatocyte diameter under the conditions described in the Section “Materials and Methods” (only hepatocytes with an evident nucleus were considered for the morphometric study). According to the results, hepatocytes had a constant diameter under AL conditions (~20.5 μm) (Figure 4C). DRF treatment promoted fluctuations in hepatocyte diameter: 12% decrease at 11:00 hours, a subsequent increase at 14:00 hours similar to the diameters found in the AL group, and finally a 5% decrease at 02:00 hours (Figure 4C). In addition, the Fa group showed a 23% reduction in comparison to the DRF group at 11:00 hours, whereas the Rf group presented only a 12% reduction compared to the DRF group at 14:00 hours (Figure 4C). Hepatocyte diameters in the Fa and Rf groups differed significantly.

### The Light to Dark Transition Changed pSer33 β-Catenin from the Cytosol to the Plasma Membrane

**Figure 5:**
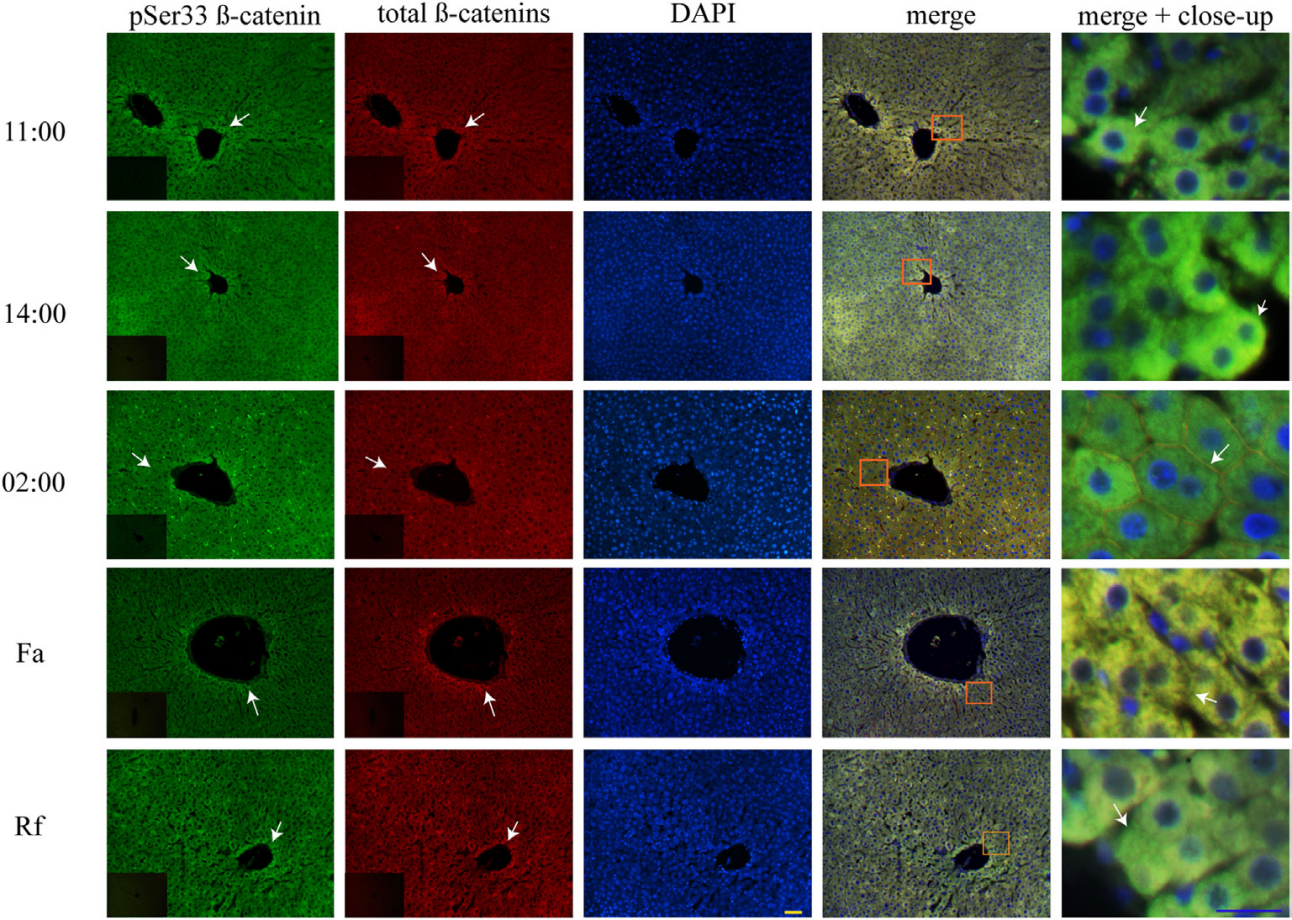
**Cytosolic co-localization of pSer33 β-catenin and total β-catenins in histological liver samples of rats under daytime restricted feeding (DRF) protocol**. Immunofluorescence signal for the pSer33 β-catenin (green), the total β-catenins (red), and the DAPI (blue) in DRF conditions at three different temporal points: 11:00, 14:00, and 02:00 hours (see Figure 4 for explanation), as well as Fa and Rf conditions. Both a panoramic (yellow calibration bar = 50 µm) and a close-up view (blue calibration bar = 25 µm) indicated by an orange square are shown. Negative controls (primary antibody omitted) are displayed in the insert of the panoramic panels. Histological liver samples from three different animals.

At two temporal points of the light phase (11:00 and 14:00 hours), both the AL and the DRF groups showed co-localization of total β-catenins and pSer33 β-catenin mainly in the cytosolic compartment of the hepatocytes (Figure S1A in Supplementary Material; Figure 5). Strikingly, the intracellular distribution of both forms of β-catenin changed in the dark phase (02:00 hours) since total β-catenins and pSer33 β-catenin were observed presumably next to the plasma membrane (Figure S1A Supplementary Material; Figure 5). Both total β-catenins and pSer33 β-catenin were still co-localized.

#### The Acute Fa–Rf Cycle Promoted pSer33 β-Catenin Location at the Hepatocyte Plasma Membrane

Unlike the AL and DRF groups, pSer33 β-catenin in Fa and Rf groups co-localized slightly with total β-catenins in the hepatocyte cytoplasm (Figure 5). Nevertheless, acute fasting and refeeding favored the location of pSer33 β-catenin next to the plasma membrane (Figure 5).

### DRF Reduced pSer675 β-Catenin Located in the Plasma Membrane

**Figure 6:**
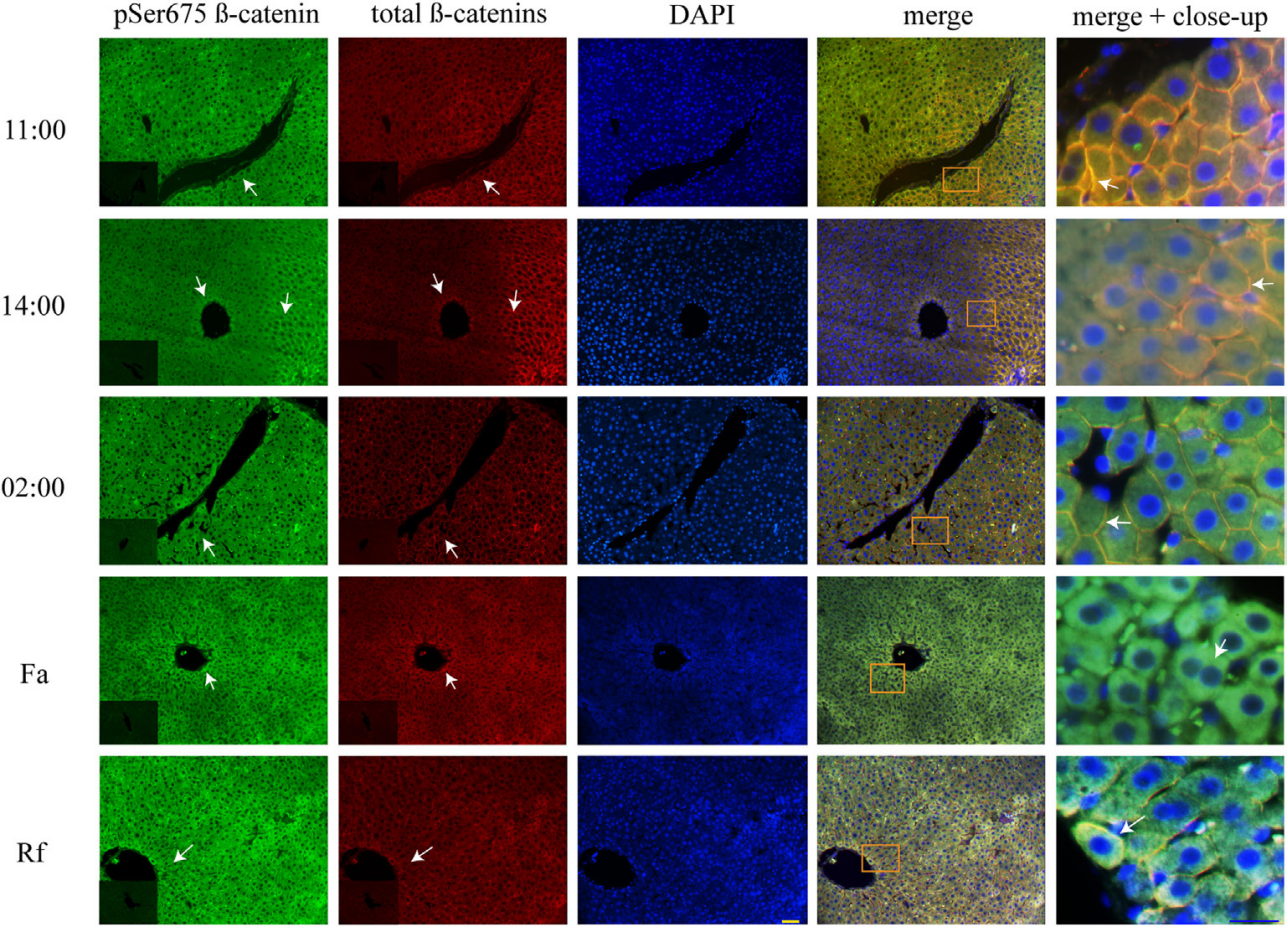
**Plasma membrane co-localization of total β-catenins and pSer675 β-catenin in histological liver samples of rats under daytime restricted feeding (DRF) protocol**. Immunofluorescence signal for pSer675 β-catenin (green), total β-catenins (red), and DAPI (blue) under DRF conditions at three different temporal points: 11:00, 14:00, and 02:00 hours (see Figure 4, for explanation), as well as under Fa and Rf conditions. Both a panoramic (yellow calibration bar = 50 µm) and a close-up view (blue calibration bar = 25 µm) indicated by an orange square are shown. Negative controls (primary antibody omitted) are displayed in the insert of the panoramic panels. Histological liver samples from three different animals.

Double immunohistochemistry of pSer675 β-catenin and total β-catenins proteins was performed to learn about pSer675 β-catenin’s subcellular distribution, response to feeding protocols (DRF and Fa–Rf), and co-localization with total β-catenins. Results revealed plasma membrane distribution under AL (Figure S2 in Supplementary Material) and DRF conditions (Figure 6) at 11:00, 14:00, and 02:00 hours. Co-localization with total β-catenins were observed at these 3 h. However, after DRF rats had access to food (at 14:00 hours), they also exhibited pSer675 β-catenin with cytosolic distribution around the vasculature. Although AL and DRF groups exhibited pSer675 β-catenin in the plasma membrane (Figure 6), the average DRF values were 43% lower than the average AL values (Figure S2B in Supplementary Material). Conversely, while the Fa group showed a presence of pSer675 β-catenin in cytosol, a relocation of this phosphorylated form of β-catenin was observed close to the plasma membrane in the Rf group. Both feeding condition groups expressed co-localization with total β-catenins (Figure 6).

### Liver Zonation of β-Catenin Variants

**Figure 7:**
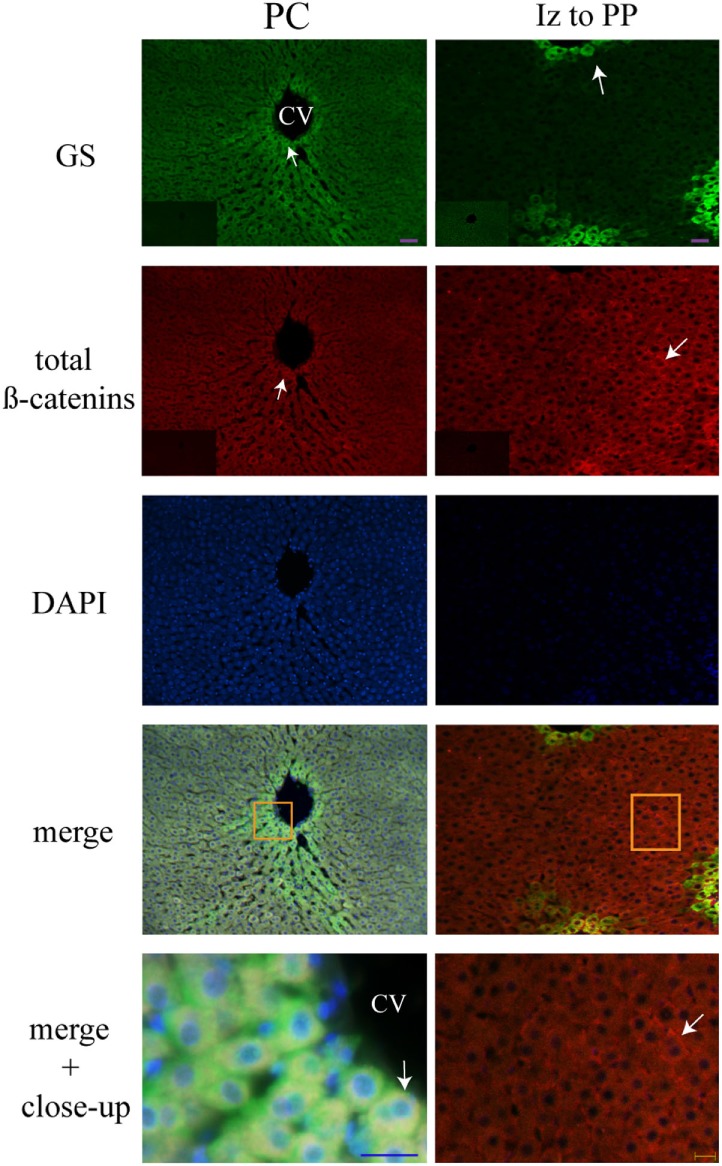
**Zonal distribution of total β-catenins in the hepatic acinus**. Immunofluorescence signal for glutamine synthetase (green), total β-catenins (red), and DAPI (blue). Both a panoramic (purple calibration bar = 50 µm) and a close-up view (blue calibration bar = 25 µm and yellow calibration bar = 20 µm) indicated by an orange square are shown. Negative controls (primary antibody omitted) are displayed in the insert of the panoramic panels. Histological liver samples from three different animals. PC, pericentral zone; Iz, intermediate zone; PP, periportal zone; CV, central vein.

**Figure 8:**
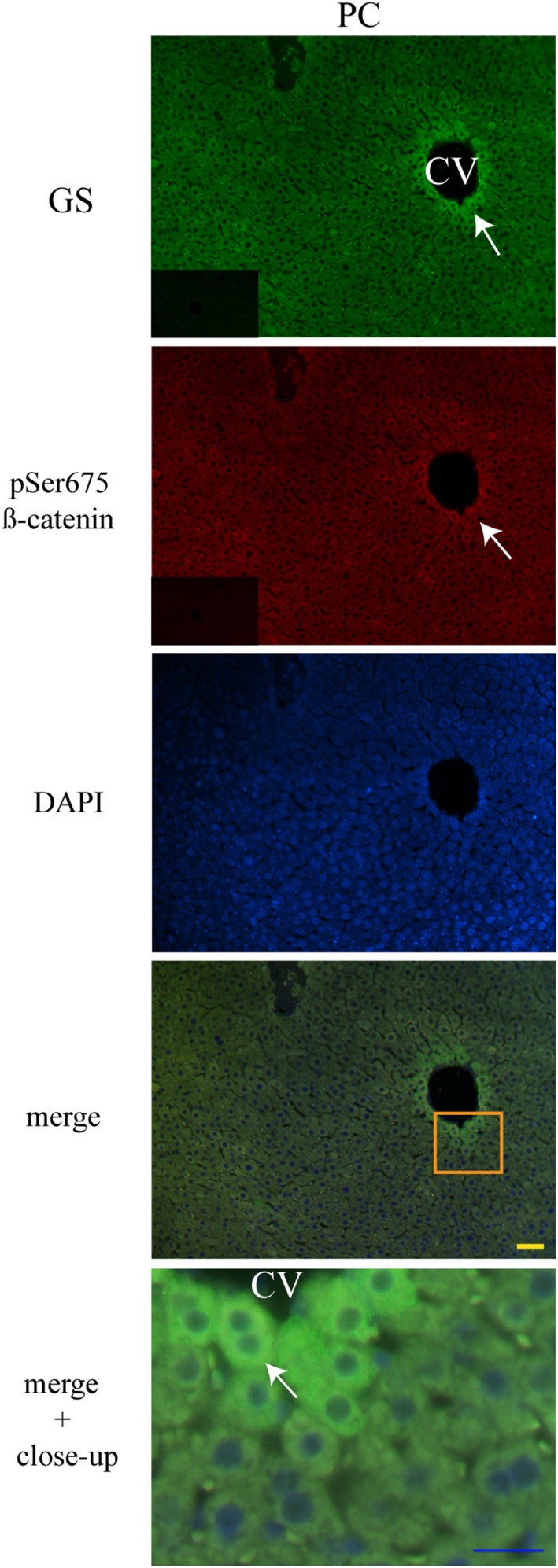
**Zonal distribution of pSer675 β-catenin in the hepatic acinus**. Immunofluorescence signal for glutamine synthetase (green), pSer675 β-catenin (red), and DAPI (blue). Both a panoramic (yellow calibration bar = 50 µm) and a close-up view (blue calibration bar = 25 µm) indicated by an orange square are shown. Negative controls (primary antibody omitted) are displayed in the insert of the panoramic panels. Histological liver samples from three different animals. CV = central vein.

**Figure 9:**
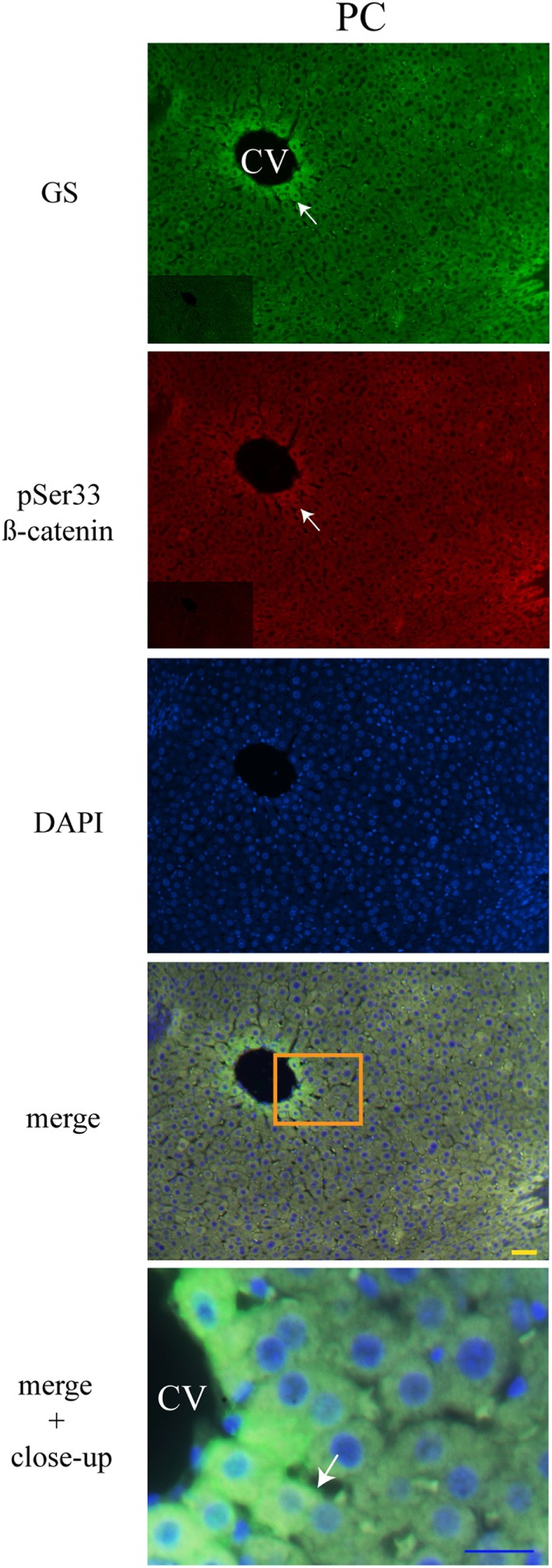
**Zonal distribution of pSer33 β-catenin in the hepatic acinus**. Immunofluorescence signal for glutamine synthase (green), pSer33 β-catenin (red), and DAPI (blue). Both a panoramic (yellow calibration bar = 50 µm) and a close-up view (blue calibration bar = 25 µm) indicated by an orange square are shown. Negative controls (primary antibody omitted) are displayed in the insert of the panoramic panels. Histological liver samples from 3 different animals. PC = pericentral zone; CV = central vein.

The three forms of β-catenin (total β-catenin, pSer33 β-catenin, and pSer675 β-catenin) displayed distinctive subcellular expressions influenced by the time in the 24-h cycle and the feeding condition. Previous reports have shown that the expression of different forms of β-catenin vary in the pericentral (PC) and periportal (PP) hepatocytes in the hepatic lobule (44, 45). Therefore, to explore a putative enrichment in the presence of β-catenin forms in the PP and PC hepatocyte population, a double immunohistochemistry was performed on all β-catenin proteins, and the GS enzyme, the canonical marker of the PC zone of the hepatic acinus. Results exhibited that total β-catenins and the pSer675 β-catenin in all schedules and conditions (11:00, 14:00, and 02:00 hours and Fa and Rf) were located mostly in the cytosol of PC hepatocytes, while from the intermediate to the PP zone of acinus, they were located in the plasma membrane (Figures 7 and 8, respectively). On the other hand, the presence of both cytosolic and cell membrane pSer33 β-catenin was observed in PC hepatocytes (Figure 9); this presence disappears between the intermediate and PP zones. That was observed in all schedules and conditions proven (11:00, 14:00, and 02:00 hours and Fa and Rf).

## Discussion

### Cell Biology and β-Catenin Signaling

Wnt/β-catenin is a conserved signaling pathway (46) that fulfills important metabolic roles in the adult liver. β-catenin plays a pivotal role that involves cell adhesion (a stable β-catenin pool associated with the cell membrane) and transcriptional activity (a soluble β-catenin cytoplasmic pool) (47). These roles are coordinated by PTMs, mainly phosphorylation (48).

Our results indicated a significant decrease of pSer33 β-catenin in the total homogenate and in the cytosol under DRF, which could indicate a lower rate for protein degradation and an opportunity for β-catenin to be translocated into the nucleus to promote transcription of its target genes.

Although the GSK3β phosphorylates β-catenin in the serine 33, this form of β-catenin did not show a rhythmic patron (Figures 1A,B). Presumably, this is because multiple and consecutive phosphorylation events are required. First, it is necessary that CK1α and GSK3β hyperphosphorylate the scaffold proteins of the destruction complex (APC and axin), which increase their affinity for β-catenin. Once the axin–APC–β-catenin complex is formed, CK1 phosphorylates the β-catenin in the serine 45; IkB kinase-α (49) and cyclin D1/Cdk6 (50) can also phosphorylate this site. This priming phosphorylation subsequently promotes that GSK3β phosphorylates threonine 41 and serines 37, and 33 (51), and these residues can be also phosphorylated by PKC (52). In this context, the protein phosphatase 1 acts on axin (53), and in consequence, GSK3β function is impeded. Also, in epithelial cells, β-catenin is constitutive synthesized to form AJ with the E-cadherin (54). Cytosolic presence of newly synthesized β-catenin and release of β-catenin from AJ are regulated by the destruction complex. Thus, we hypothesize that multifactorial equilibrium between phosphorylation and dephosphorylation events by a variety of cellular inputs maintains constant pSer33 β-catenin expression levels.

Differences among pSer33 β-catenin temporal points in the nucleus (Figure 1C) could be explained by the fact that pSer33 β-catenin is not transcriptionally active; consequently, it could be periodically exported from the cell nucleus.

Concerning the pSer675 β-catenin expression, AL group presented substantial changes in the night, where normally animals eat, and metabolic parameters such as glycemia and insulin are increased (18), a pattern expected for species with nocturnal feeding habits. In the case of DRF animals, pSer675 β-catenin expression was constant in all fractions, but it increased in nucleus (Figure 2C). It is known that DRF animals are hypoglycemic and they present high levels of corticosterone and glucagon as well as low levels of insulin (18), three positive regulators of GNG.

Protein kinase A, which phosphorylates β-catenin at serine 675 (26), is stimulated by glucagon. This condition could be related to the pSer675 β-catenin transcriptional activity associated with repeated fasting and the consequent GNG activation. GNG is a physiological adaptation to starvation (short and medium fasting), stressful conditions, and short-term glycogen-replenishment activity in the liver after food intake (18). It has been confirmed that in the liver of starved animals, β-catenin regulates hepatic glucose metabolism through transcriptional regulation of cyclin D1, which controls the gluconeogenic response in addition to its role in the cell cycle (55, 56). Furthermore, β-catenin regulates the transcription of two of the rate-limiting enzymes in hepatic GNG: glucose-6-phosphatase (G6Pase) and phosphoenolpyruvate carboxykinase (PEPCK) by interacting with the FoxO1 transcription factor (22).

Indeed, GNG also increases under Fa and Rf conditions. However, when compared to DRF, Fa rats showed a higher presence of pSer675 in the cytosolic fraction (Figure 2B), which could be related to a synergic regulation by other signaling pathway elements such as Akt (57), glucagon-like peptide 1 (57), glucagon-like peptide 2 (58), p21 (27), or insulin growth factor 1 (59).

Substantial differences between DRF protocol and an acute 22 h fasting (Fa) are presented in a diversity of metabolic parameters (15, 17–19, 38, 60, 61), supporting the notion that the metabolic state of rats under DRF protocol is unique and distinct from fed and fasted animals. It is proposed under DRF protocol that hepatic physiology acquires a rheostatic state (12, 17) as a result of biochemical and physiological adaptations for a better handling of nutrients.

It has been also reported that the amount of nuclear active dephosphorylated form of β-catenin increases after an overnight fast (22), which could be probably related to the significant incremented expression of total β-catenins in the cytosol and in the cell nucleus under DRF, Fa, and Rf conditions as a response to both acute and repeated fasting (Figure 3). DRF shifted the peaks of PEPCK and G6Pase around the time of food access (18), which coincides with the increase of total β-catenins mainly in the light phase (Figure 3). DRF also enhanced hepatic PEPCK activity and increased the amount of hepatic G6Pase (18). Therefore, we propose that elevated levels of total β-catenins within the nucleus could exert transcriptional activity, and the increased expression of total β-catenins in the plasma membrane (Figures 4A,B) under DRF could be related to cell adhesion properties. β-catenin can bind to type I cadherins, and it plays an essential role in the structural organization of tissues forming the cadherin–catenin complex, which is the base of AJ, to establish and maintain epithelial polarity (62). In hepatocytes, the polarization arrangement is unique and contributes to form the bile canaliculus, the smallest branch of the bile duct that forms a complex interconnected network that spreads along the liver parenchyma, in which tight junctions (TJ) are essential (63). By using a microarray technique, our group detected that, under DRF conditions, both cadherin (AJ) and claudin (TJ) increased by 13.3- and 6.4-folds, respectively, (data not published) after food intake at 14:00 hours; this temporal point is coincident with the maximal expression of total β-catenins in the plasma membrane (Figure 4B). At 14:00 hours, hepatocytes showed a larger diameter in comparison to the other schedules and conditions. This fact exhibits a correlation between total β-catenins presence and proteins related to hepatocyte morphology. Díaz-Muñoz et al. (19) demonstrated that DRF can modify the cross-sectional area of hepatocytes during FAA because at 11:00 hours hepatocytes were about 53% larger than under DRF before (08:00 hours) and after FAA (14:00 hours). In addition, Steinberg and Takeichi (64) postulated that the expression level of adhesion molecules, such as cadherins, influences the strength of adhesion, which provides active adhesion gradients in both vertebrate and insect developing systems. These gradients can determine both morphogenetic movements and specific anatomical configurations (65). Considering that liver weight decreases (~15%) under DRF treatment mainly during the light phase (14), it is possible that the β-catenin–cadherin complex could act as a flexible cell border involved in daily cell size changes: a decrease in hepatocyte diameter after 22 h of fasting (11:00 hours) and an increase in hepatocyte diameter upon replenishing their glycogen after 2 h of food access (14:00 hours) (18) (Figure 4C). Indeed, this dynamic cycle of decrease/increase in cell proportion is more noticeable under Fa and Rf conditions (Figure 4C).

Regarding the cellular localization of the three different forms of β-catenin, Benhamouche et al. (44) demonstrated both a robust β-catenin expression in the hepatocyte membrane (related to cell adhesion) and a slight cytosolic accumulation of β-catenin in the PC zone of hepatic lobule, while in PP hepatocytes staining in hepatocyte membrane decreased. When they probed the unphosphorylated form of β-catenin (an active form of protein), localization was cytosolic in the both proximal and distal part of PC compartment, whereas its negative regulator APC was expressed in the PP zone. This complementary distribution of β-catenin and APC suggests opposite effects of β-catenin pathway along hepatic acinus.

We observed that pSer33 β-catenin is present in the cytosol mainly at 11:00 and 14:00 hours and in the plasma membrane at 02:00 hours, both under DRF (Figure 5) and AL (Figure S1 in Supplementary Material) conditions; probably to a daily target for β-catenin degradation that is more evident during the light phase. According to western blot results, β-catenin marked for degradation in the Fa and Rf groups is not as noticeable as the other two groups (Figure 1B). Presumably, pSer33 β-catenin localization in the plasma membrane is due to a dynamic equilibrium between phosphorylation and dephosphorylation. Unexpectedly, we found pSer675 β-catenin close to the plasma membrane (Figure 6; Figure S2A in Supplementary Material). Semiquantification of pSer675 at the hepatocyte periphery under AL and DRF conditions demonstrated a decreased average in the DRF group in comparison to the AL group (Figure S2B in Supplementary Material). Apparently, the above result indicate that pSer675 β-catenin under DRF could be acting as a reservoir for β-catenin to eventually be translocated to the nucleus. In the Fa group, pSer675 β-catenin cytosolic expression (Figure 6) correlates with western blot results (Figure 2B), probably due to further phosphorylation associated with other signaling pathways.

With respect to the localization of β-catenin pools in the hepatic acinus, these showed cytosolic distribution in the PC zone, whereas detection in the plasma membrane was evident from the intermediate to the PP zones (Figures 6–8). Benhamouche et al.’s report in 2006 established a key role of Wnt/β-catenin pathway in liver zonation, which entails a functional and structural cellular heterogeneity. This hepatic zonation suggests an anatomical specialization in β-catenin functions, where in the PC zone, β-catenin that is committed to cell signaling could be accumulated in the cytosol waiting to be shuttled into the nucleus or to be degraded, whereas in the PP zone, β-catenin that plays a structural/reservoir role could be accumulated in the plasma membrane. Recently, the molecular mechanism that controls metabolic liver zonation was determined (66), showing complexity of this phenomena, where the APC protein and the RSPO-LGR4/5-ZNRF3/RNF43 module play essential and complementary roles.

**Figure 10:**
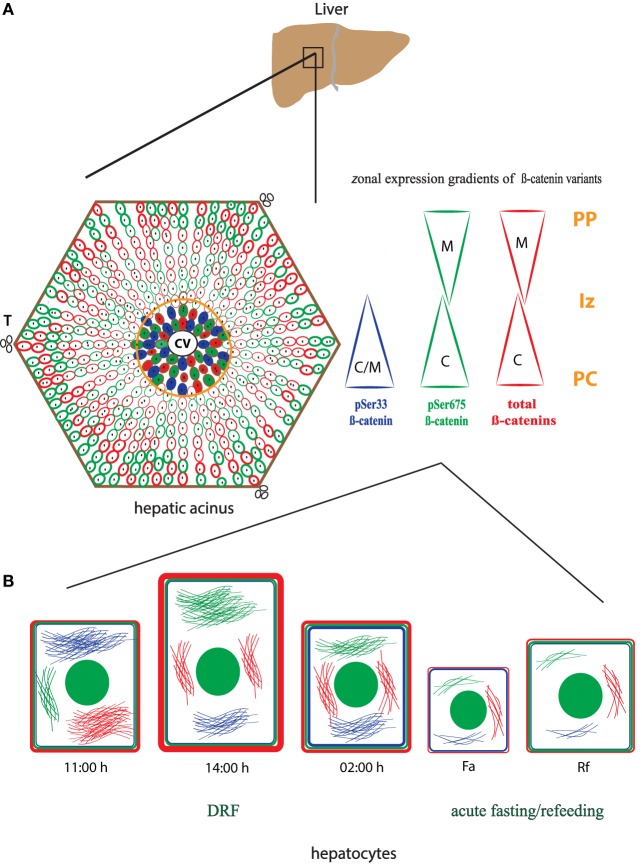
**Schematic model of the main changes and adaptations showed by total β-catenins and its phosphorylated forms: pSer33 and p675 in the hepatic acinus under daytime restricted feeding protocol**. **(A)** Zonal localization of the three different pools of β-catenin studied, total β-catenins (red), pSer33 β-catenin (blue), and p675 β-catenin (green) within the functional unit of liver, the hepatic acinus. This localization is represented in the form of gradients (triangles) according to the distinct zones of the acinus. PC was signaled and delimited by an orange circle. **(B)** Intracellular and plasma membrane localization of the β-catenin pools mentioned in **(A)** in hepatocytes at 11:00, 14:00, and 02:00 hours, as well as under Fa and Rf conditions. Hepatocytes size varies according to the time of the day and the feeding condition. Color filaments represent cytosolic localization of distinct β-catenins, and the green circle inside hepatocytes represent cell nucleus. CV, central vein; T, portal triad; PC, pericentral zone; Iz, intermediate zone; PP, periportal zone; C, cytosolic expression; M, plasma membrane expression.

Our results indicate that β-catenin is sensitive to feeding conditions and that both its structural and transcriptional functions are differentially modulated by the DRF protocol. These findings are significant because it would establish β-catenin as an element of the liver metabolic network that is closely related to the circadian molecular clock. To integrate our western blot and immunohistochemical results of the three forms of β-catenin, we elaborated a dynamic model to depict the liver response and adaptation to 2 h of DRF protocol at different times of the day (11:00, 14:00, and 02:00 hours) and feeding conditions (Fa and Rf) (Figure 10). In this model, we represented the gradient in the expression of the three different pools of β-catenin studied (total β-catenins, pSer33 β-catenin, and pSer675 β-catenin), along the hepatic acinus [metabolic zonation from the central vein (CV) to the portal triad (T)] (Figure 10A). The intracellular location of each β-catenin within the hepatocyte, besides the hepatocyte morphometry (Figure 10B), is shown. The cytoplasmic location of the three β-catenins in the PC hepatocytes can be seen, whereas the presence of total β-catenins and pSer675 in the PP hepatocytes is mostly in the plasma membrane region.

### The β-Catenin Protein and FEO Expression

The core of liver circadian clock is based on a molecular mechanism that includes transcriptional/translational loops (67). In this mechanism, we found positive regulators such as the BMAL1 protein and negative regulators such as the PER protein. Both BMAL1 and PER1 show circadian rhythmicity in the liver (68), whose acrophases are modified by the DRF protocol (14). In this sense, experiments with cell cultures of NIH-3T3 cells have shown that overexpressions of BMAL1 increase β-catenin mRNA levels, indicating a direct relationship between them (69). In addition, downregulation of PER2 increases β-catenin in human colon cancer cells (70) and in the breast cancer cell line (MTCL) (30). These data suggest that clock genes in peripheral tissues regulate β-catenin expression. In the liver and other organs, DRF protocol promotes metabolic adaptations and changes in the daily rhythmicity of a various proteins and enzymes from different metabolic pathways (18), hormones (14, 16), receptors (15), calcium dynamics (38), and nuclear receptors (71). Liu et al. (22) demonstrated that β-catenin in the liver acts as a regulator of circulating glucose, GNG and as a modulator of insulin signaling. Moreover, it plays a role in mitochondrial homeostasis and, consequently, in energy balance (72). Many of these metabolic functions show a daily rhythmicity that is affected by the DRF protocol.

In mammals, the suprachiasmatic nuclei (SCN) of the hypothalamus is the main circadian pacemaker (68, 73), which is synchronized by light–dark cycle and organizes the timing of peripheral organs through neural and hormonal pathways (74). The core of circadian system is based on a transcriptional–translational feedback loops of genes and proteins whose oscillating period is near 24 h. Besides light cues, other environmental factors as periodic feeding can entrain circadian rhythms in peripheral oscillators as the liver. In this sense, when food availability is restricted 2–3 h per day (DRF), a new configuration of circadian system called FEO emerges. The most evident behavior associated to DRF in rodents is the FAA, which persists even when the SCN has been completed ablated (75) and for instance is considered the result of a circadian oscillator different to SCN.

DRF protocol implies both timed restricted feeding and calorie restriction to foster a generalized anticipatory state that optimizes searching, assimilation, and processing of nutrients. In peripheral oscillators, adaptations to restricted feeding paradigm includes a shift in the acrophase of clock genes, hormones, and many proteins (76–78) involved in metabolic pathways near the time of food accessibility. Under a DRF protocol, we found many interaction points between the molecular circadian clock and the metabolism that are under chronostatic regulation (12). Although the anatomical substrate of the FEO is unknown yet, the liver acts in a coordinated manner with other peripheral oscillators to induce an anticipatory behavior to prepare the animal to search for food.

Due to only a couple hours of food access in the DRF protocol, experimental animals consume lesser quantity of food (~36%) and reduce their body weight (~28%) (data not shown) and nutritional conditions change to be hypocaloric (79) compared to AL group. Calorie restriction involves a reduction in caloric intake (24–60%) from the macronutrients respect to AL animals (80). Animal below hypocaloric conditions did not present malnutrition (81), which had been corroborated in our laboratory by parameters such as albumin, hemoglobin concentration, and mean corpuscular hemoglobin (data not shown). Importance of calorie restriction combined or not with a DRF protocol resides in the resetting of the SCN (82), promoting interaction between the master circadian clock and metabolism. Moreover, the cross-talk between the circadian clock and Wnt signaling relies on two kinases: CK1 and GSK3β. These kinases phosphorylate clock proteins contributing to the fine-tuning regulation of the circadian clock (83), and they downregulate β-catenin activity in the Wnt/β-catenin pathway (84). The interactions between clock proteins, β-catenin, and different kinases could be a robust switch that coordinates metabolic changes in the liver under the DRF protocol. Further experiments are needed to elucidate if β-catenin, as a structural or as a signaling element, could be acting as an output factor of the hepatic circadian molecular clock.

### Implications of β-Catenin in Liver Disease

β-catenin is a transcription factor for cell cycle regulators such as cyclin D1 (55) and c-myc (85). Moreover, β-catenin is essential in liver physiology (86). Aberrant β-catenin pathway activation has been associated with various liver pathologies such as defective bile acid metabolism, hepatosteatosis and cholestasis (87), chronic liver disease, hepatic fibrosis, and hepatocellular cancer (HCC) (88). In this sense, it has been reported that HCC cells from animal models and human patients show a constitutive activation of Wnt/β-catenin signaling (89). In addition, PER and BMAL1 could influence cell proliferation through Wnt pathway activation (69). It has also been proposed that cancer may be a circadian-related disorder (90) and that DRF decreases tumor size in HCC induced by dimethylnitrosamine (data not shown). All these data strengthen the idea that β-catenin could be considered a key factor in future therapeutic strategies for various liver pathologies.

In conclusion, our results suggest that β-catenin is an essential element in the metabolism and circadian context of liver physiology. Interestingly, β-catenin functions in the liver are zonated, which provides versatility for a good adaptation to metabolic challenges. Further studies are needed to define the mechanistic relationship between these findings and the FEO expression.

## Author Contributions

DI-P and MD-M designed the project and wrote the manuscript. DI-P was responsible for the acquisition, analysis of data, and the interpretation of experimental work. Both authors approved the final manuscript for publication and agreed to be accountable for all aspects of the work presented therein.

## Conflict of Interest Statement

The authors declare that the research was conducted in the absence of any commercial or financial relationships that could be construed as a potential conflict of interest.

## Supplementary Material

Figure S1 **Cytosolic co-localization of pSer33 β-catenin and total β-catenins in histological liver samples of AL rats**. Immunofluorescence signal for pSer33 β-catenin (green), total β-catenins (red), and DAPI (blue) under AL conditions at three different temporal points: 11:00, 14:00, and 02:00 hours (see Figure 4, for explanation). Both a panoramic (yellow calibration bar = 50 µm) and a close-up view (blue calibration bar = 25 µm) indicated by an orange square are shown. Negative controls (primary antibody omitted) are displayed in the insert of the panoramic panels. Histological liver samples from three different animals.

Figure S2 **Plasma membrane co-localization of total β-catenins and pSer675 β-catenin in histological liver samples of AL rats**. **(A)** Immunofluorescence signal for pSer675 β-catenin (green), total β-catenins (red), and DAPI (blue) under AL conditions at three different temporal points: 11:00, 14:00, and 02:00 hours (see Figure 4, for explanation). Both a panoramic (yellow calibration bar = 50 µm) and a close-up view (blue calibration bar = 25 µm) indicated by an orange square are shown. Negative controls (primary antibody omitted) are displayed in the insert of the panoramic panels. Histological liver samples from three different animals. Histogram showing quantification of **(B)** plasma membrane presence of pSer675 β-catenin in hepatocytes (*n* = 3 animals) at same temporal points described above under AL (white bars), DRF (black bars), and Fa and Rf (gray bars). Data are represented as the mean ± SEM. The horizontal gray lines represent the schedules average of each condition. ^ψ^Significant difference between AL and DRF groups average (Student’s *t*-test, *p* < 0.05). *Significant difference between AL and DRF groups at the same temporal point (Student’s *t*-test, *p* < 0.05). f.a.u, fluorescence arbitrary units; nd, not detected.
