# Supplementary figures and images for: Synchronization by Daytime Restricted Food Access Modulates the Presence and Subcellular Distribution of β-Catenin and Its Phosphorylated Forms in the Rat Liver
Source: Front Endocrinol (Lausanne). 2017 Feb 6;8:14. doi: 10.3389/fendo.2017.00014 (PMC5292920; doi:10.3389/fendo.2017.00014)

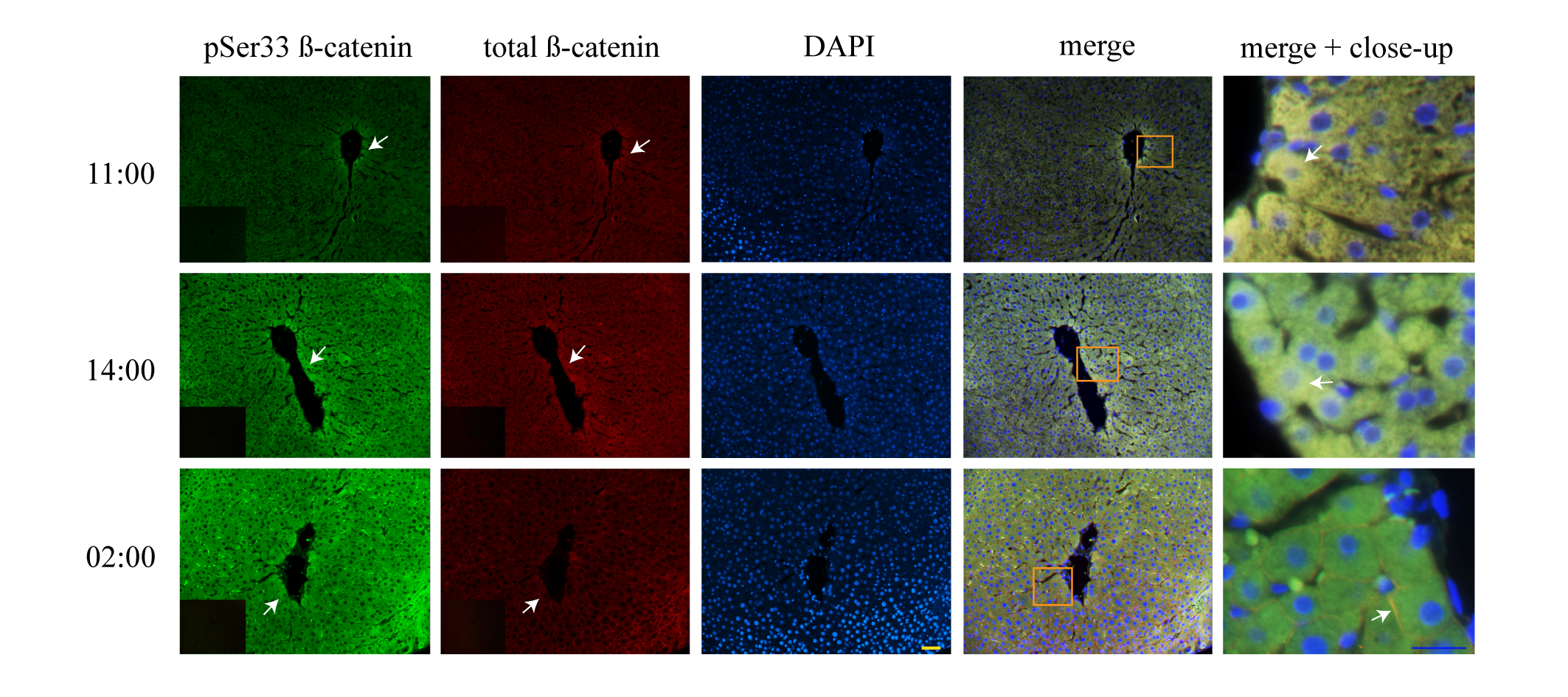

Supplement: Figure S1 — Cytosolic co-localization of pSer33 β-catenin and total β-catenins in histological liver samples of AL rats. Immunofluorescence signal for pSer33 β-catenin (green), total β-catenins (red), and DAPI (blue) under AL conditions at three different temporal points: 11:00, 14:00, and 02:00 hours (see Figure 4, for explanation). Both a panoramic (yellow calibration bar = 50 µm) and a close-up view (blue calibration bar = 25 µm) indicated by an orange square are shown. Negative controls (primary antibody omitted) are displayed in the insert of the panoramic panels. Histological liver samples from three different animals. [file image_1.tif]

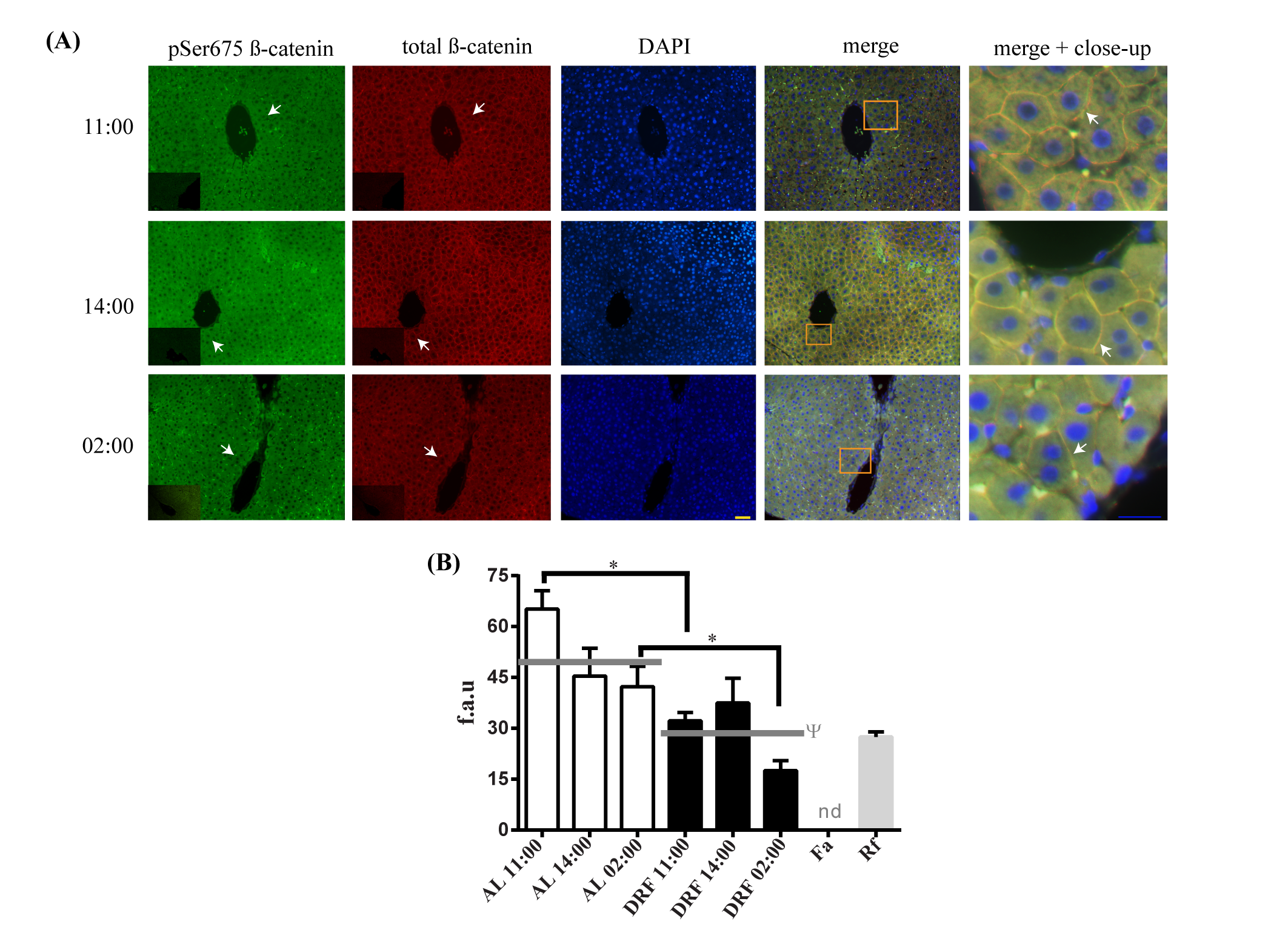

Supplement: Figure S2 — Plasma membrane co-localization of total β-catenins and pSer675 β-catenin in histological liver samples of AL rats. (A) Immunofluorescence signal for pSer675 β-catenin (green), total β-catenins (red), and DAPI (blue) under AL conditions at three different temporal points: 11:00, 14:00, and 02:00 hours (see Figure 4, for explanation). Both a panoramic (yellow calibration bar = 50 µm) and a close-up view (blue calibration bar = 25 µm) indicated by an orange square are shown. Negative controls (primary antibody omitted) are displayed in the insert of the panoramic panels. Histological liver samples from three different animals. Histogram showing quantification of (B) plasma membrane presence of pSer675 β-catenin in hepatocytes (n = 3 animals) at same temporal points described above under AL (white bars), DRF (black bars), and Fa and Rf (gray bars). Data are represented as the mean ± SEM. The horizontal gray lines represent the schedules average of each condition. ψSignificant difference between AL and DRF groups average (Student’s t-test, p < 0.05). *Significant difference between AL and DRF groups at the same temporal point (Student’s t-test, p < 0.05). f.a.u, fluorescence arbitrary units; nd, not detected. [file image_2.tif]
